# Multifunctional Nanofiber Composites Integrating pH‐Responsiveness and Passive Daytime Radiative Cooling

**DOI:** 10.1002/nap2.70036

**Published:** 2026-04-01

**Authors:** Seokgyu Kwon, Minseo Jeong, Changhwan Hyeon, Juhoon Baek, Minkyung Kim, Dasol Lee

**Affiliations:** ^1^ Department of Biomedical Engineering Yonsei University Wonju Republic of Korea; ^2^ Department of Mechanical and Robotics Engineering Gwangju Institute of Science and Technology (GIST) Gwangju Republic of Korea

**Keywords:** electrospinning, nanofiber composites, pH‐responsiveness, radiative cooling

## Abstract

Nanofiber composites integrating passive daytime radiative cooling with pH‐responsiveness were developed using polyvinyl alcohol (PVA), lignin, and aluminum oxide. The composite structure was engineered to combine high solar reflectance, strong emissivity within the atmospheric window (8–13 μm), and reversible swelling behavior in response to pH variation. Aluminum oxide nanoparticles enhance solar reflectance, whereas PVA and lignin provide functional groups enabling the formation of pH‐sensitive hydrogel networks. Based on these functional groups, citric acid was introduced as a crosslinking agent to form ester bonds, thereby enhancing the mechanical strength and structural stability of the composite under aqueous conditions. The fabricated nanofiber composite exhibited excellent radiative cooling performance, characterized by high solar reflectance and strong mid‐infrared emissivity. Moreover, even in the state of hydrogel, the material maintained stable radiative cooling capability under different pH environments. The independent operation of radiative cooling and pH‐responsiveness enables application in diverse scenarios where passive heat dissipation and environmental sensing are both advantageous. Potential fields include biomedical systems, smart packaging, and responsive textiles, where adaptive functionality can address dynamic environmental demands. This study establishes a design strategy for multifunctional nanofiber composites that extend radiative cooling technologies into stimuli‐responsive domains.

## Graphical abstract

This study introduces multifunctional nanofiber composites integrating radiative cooling (RC) and pH‐responsiveness. The composites were demonstrated to exhibit high solar reflectance, strong MIR emissivity, cooling performance under outdoor conditions, and RC capability even in hydrogel states across different pH environments. This study provides a foundation for extending RC technologies beyond static thermal regulation toward dynamically responsive applications.

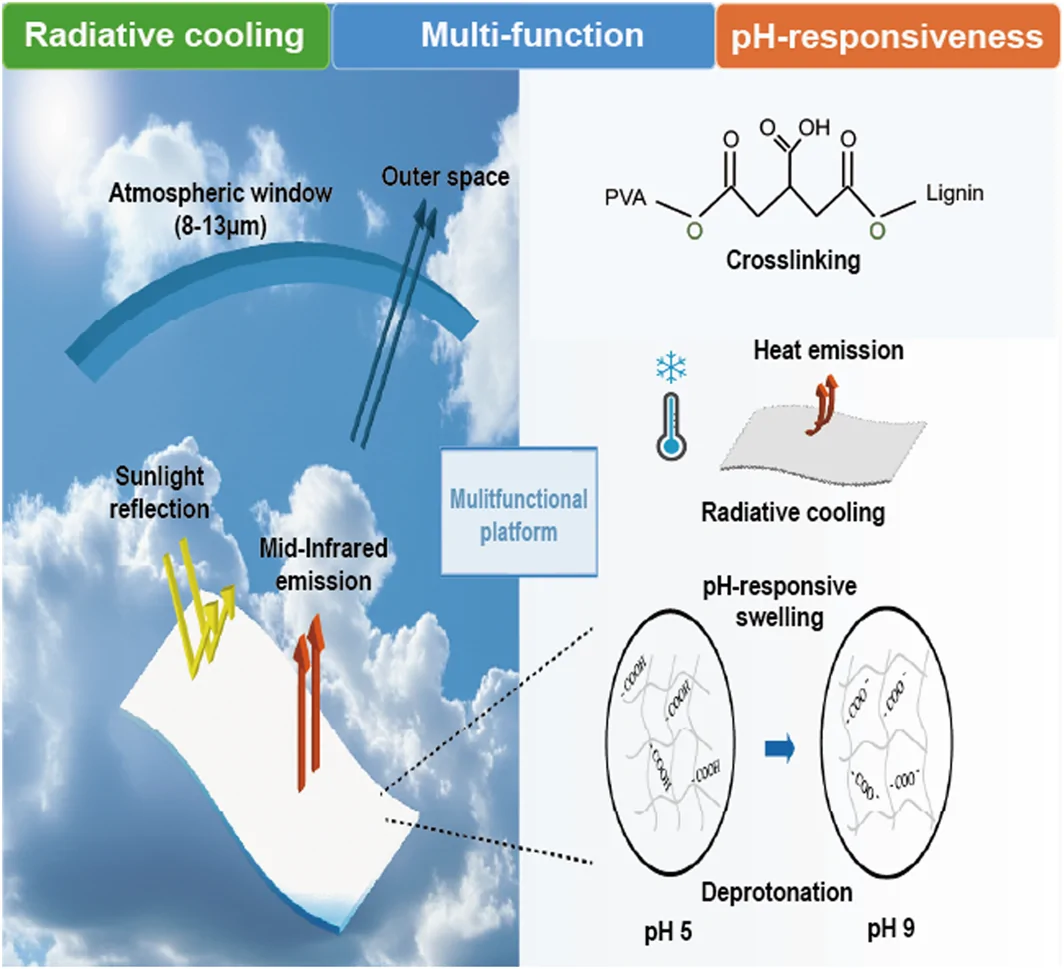

## 1 Introduction

With the acceleration of global warming, the significance and necessity of passive daytime radiative cooling technologies are increasingly recognized [1, 2, 3, 4, 5, 6]. Radiative cooling (RC) is a technique that enables surfaces to reduce their temperature without energy consumption by combining high reflectance in the solar spectrum and emissivity in the mid‐infrared (MIR) region. In particular, the atmospheric window (AW) (8–13 μm) allows thermal radiation from the object to escape directly into outer space. By maximizing emissivity within this spectral window while minimizing solar absorption, RC materials can effectively dissipate heat to the cold universe, thereby achieving a net cooling effect below ambient air temperature. These spectral characteristics form the basis for material design strategies in RC applications [7, 8, 9, 10]. In response to this growing demand, RC has been used in various applications, including device thermal management [11, 12, 13, 14], energy harvesting [15, 16, 17], and building temperature regulation [18, 19].

Despite increasing interest in RC technologies, their implementation has largely remained within structural or large‐scale thermal management systems. However, many real‐world environments require not only passive thermal regulation but also responsiveness to environmental stimuli, such as pH [20, 21, 22, 23] or electric fields [24, 25]. Integrating such stimulus‐responsive functionalities into RC materials could expand their applicability to broader contexts where dynamic environmental adaptation is beneficial. Among these, pH responsiveness is particularly promising, as local pH variations are often associated with chemical or biological changes. This capability may enable new functionalities in RC materials, particularly in fields such as biomedical systems [26, 27], smart textiles [28, 29], or packaging [30, 31], where both thermal regulation and chemical sensitivity are desired. To achieve this, our study introduces a composite material developed by integrating polyvinyl alcohol (PVA), lignin, and aluminum oxide (Al_2_O_3_). This multifunctional platform exhibits both passive cooling and pH‐responsive functionality, distinguishing it from materials designed for simple thermal regulation.

PVA is a low‐cost, biocompatible polymer with strong MIR vibrational modes that support high emissivity in the AW [32, 33]. Among sustainable RC materials, biomass‐based candidates such as cellulose and wood have recently attracted attention as environmentally friendly alternatives. However, their processing often presents challenges. For instance, despite recent advances in green solvents, fabricating cellulose nanofibers typically necessitates complex multi‐step regeneration processes or precise dissolution control due to its inherent recalcitrance [34, 35], whereas the inherent rigidity of natural wood limits the utility of wood‐based coolers on curved or flexible surfaces [36, 37]. In contrast, PVA offers distinct advantages; it allows for easy processing using water, exhibits good electrospinnability for continuous nanofiber fabrication, and possesses excellent mechanical flexibility. Complementing this PVA matrix, lignin is a renewable biopolymer whose ionizable groups enable pH‐responsive behavior and additionally enhance MIR emission [38, 39, 40, 41]. In addition, the abundant hydroxyl and carboxyl groups in both PVA and lignin enable physical or chemical crosslinking, facilitating the formation of hydrogel networks [42, 43]. This capability not only enhances their compatibility as a matrix for nanocomposites but also provides a versatile structural framework for integrating RC with pH‐responsive functionality. Although lignin inherently exhibits solar absorption due to its aromatic structure, its utilization is compelling given its unique capability to confer pH‐responsiveness and environmental sustainability. However, lignin‐based RC reported so far has dark coloration, which significantly suppresses solar reflectance in the ultraviolet (UV)—visible (VIS)—near infrared (NIR) range [41]. To overcome this limitation, this study integrates PVA and Al_2_O_3_ into a lignin‐based nanofiber system. PVA provides strong vibrational modes in the MIR region and facilitates the formation of hydrogel structures that exhibit pH‐responsive swelling behavior. Al_2_O_3_ is incorporated to further enhance reflectance in the solar spectrum range due to its dielectric behavior and high refractive index, a strategy widely adopted in previous RC studies. Moreover, Al_2_O_3_ nanoparticles enhance radiative heat dissipation by maximizing emissivity within the AW, especially 10–13 μm [44, 45, 46, 47, 48]. These material characteristics address the inherent limitation of lignin‐based RC while maintaining strong MIR emissivity and enabling pH‐responsive functionality.

Although pH‐responsive materials have been widely investigated for applications in biomedical and food packaging systems, where environmental pH fluctuations play a crucial role, such as in wound healing, infection control, and food spoilage monitoring [31, 43, 49, 50, 51], their integration with RC technologies has remained unexplored. This study fills the gap between chemical responsiveness and passive thermal management, pioneering a new materials strategy that combines RC and pH‐responsiveness within a single platform.

Therefore, we fabricated multifunctional nanofiber composites based on PVA, lignin, and Al_2_O_3_, which integrate RC and pH‐responsive properties within a single platform (Figure 1). The fabricated nanofiber composites were demonstrated to exhibit high solar reflectance, strong MIR emissivity, and stable sub‐ambient cooling performance under outdoor conditions. In addition, the material maintained its RC capability even in hydrogel states across different pH environments, thereby confirming its environmental adaptability. This study provides a foundation for extending RC technologies beyond static thermal regulation toward dynamically responsive applications.

**FIGURE 1:**
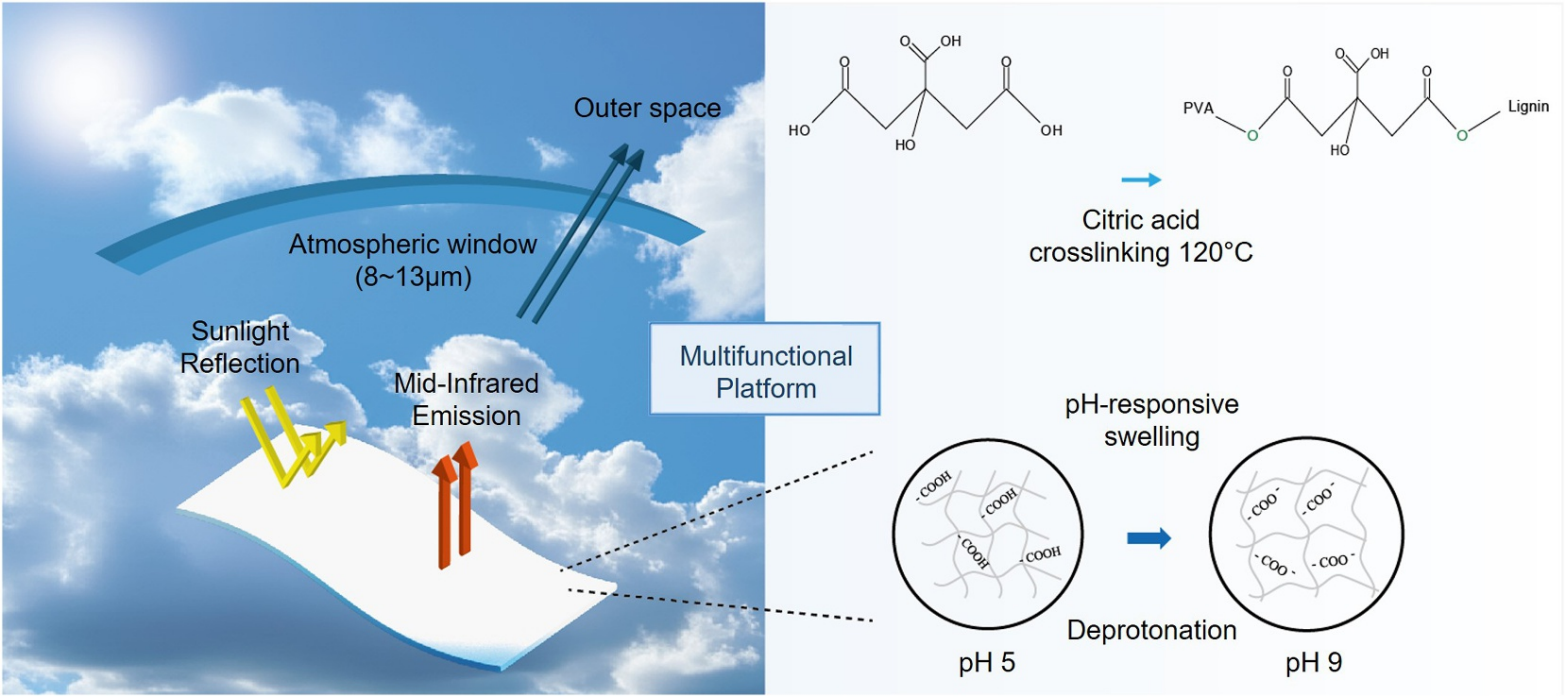
Schematic illustration of the PVA/lignin/Al_2_O_3_ nanocomposites for the multifunctional platform with pH responsive and radiative cooling.

## 2 Materials and Methods

### 2.1 Materials

Poly(vinyl alcohol) 1799 and fluorescein sodium salt (FLU) were purchased from Shanghai Aladdin Biochemical Technology Co. Ltd. Aluminum oxide nanopowder (100% alpha, no gamma, 80 nm) was purchased from US Research Nanomaterials Co. Ltd. Lignin (alkaline) was purchased from Tokyo Chemical Industry Co. Ltd. Citric acid was purchased from Wako Pure Chemical Industries Co. Ltd. Tween 20 was purchased from Junsei Co. Ltd.

### 2.2 Preparation of PVA/Lignin/Al_2_O_3_ Solutions

Firstly, a 10% (w/w) PVA solution was prepared by stirring in deionized water at 95°C for 6 h. A 1% (w/w) lignin solution was prepared similarly by stirring in deionized water at room temperature for 12 h. The PVA and lignin solutions were then mixed at a weight ratio of 9.5:0.5 and stirred at room temperature for 24 h. Subsequently, 5% (w/w) Al_2_O_3_ was stirred at room temperature for another 24 h 1% (w/w) citric acid and 0.1% (w/w) Tween 20 were added to the mixture and stirred again at room temperature for 1 h. Finally, a homogeneous solution suitable for electrospinning was obtained.

### 2.3 Preparation of PVA/Lignin/Al_2_O_3_ Solutions Containing FLU

Firstly, a 10% (w/w) PVA solution was prepared by stirring in deionized water at 95°C for 6 h. After cooling at room temperature, 0.1% (w/w) FLU was added, and the mixture was stirred until completely dissolved. 1% (w/w) lignin solution was prepared separately by stirring in deionized water at room temperature for 12 h. The PVA/FLU and lignin solutions were then mixed at a weight ratio of 9.5:0.5 and stirred at room temperature for 24 h. Subsequently, 1% (w/w) citric acid, 5% (w/w) Al_2_O_3_, and 0.1% (v/w) Tween 20 were added to the PVA/lignin mixture and stirred again at room temperature for another 24 h. Finally, a homogeneous solution suitable for electrospinning was obtained.

### 2.4 Electrospinning of PVA/Lignin/Al_2_O_3_ Nanofibers

Before electrospinning, the homogeneous solution was filled into a syringe. Electrospinning was performed at a flow rate of 0.5 mL/h under an applied voltage of 17–20 kV, with a fixed working distance of 12 cm between the needle tip and the collector. Nanofibers were collected on aluminum foil for 4 h. Subsequently, the collected fibers were placed in a drying oven at 120°C for 2 h to induce crosslinking. Finally, the PVA/lignin/Al_2_O_3_ nanofibers were successfully obtained.

### 2.5 Scanning Electron Microscopy (SEM)

The morphology of electrospun nanofibers was investigated by scanning electron microscope (SEM) (Jeol, JSM‐7500F). Fiber diameters were determined from random measurements in an SEM image using ImageJ (National Institutes of Health, Bethesda, MD, USA) [52].

### 2.6 Fourier Transform Infrared (FTIR) Characterization of Functional Groups

Spectra of the samples were measured in the spectral range between 4000 and 600 cm^− 1^, using a FTIR spectrometer (Bruker, INVENIO) in the attenuated total reflection mode, directly applying the solid film sample. The obtained spectra were further used to analyze the functional groups, their interactions between different components, and the sample's crosslinking reaction.

### 2.7 Optical Characteristics of Fabricated Nanofiber Composites

For the UV, VIS, and NIR spectral ranges (0.3–2.5 μm), the reflectance spectrum of the PVA/lignin/Al_2_O_3_ sample was measured using a UV‐Vis‐NIR spectrophotometer (Jasco, V‐770). The MIR emittance was characterized by using an FTIR spectrometer (Bruker, Vertex 70v).

Chromaticity coordinates (x, y) were obtained from sample images using ImageJ. The mean RGB values of each sample were extracted and converted to CIE XYZ values using the standard sRGB conversion matrix (illuminant D65, *γ* ≈ 2.2). The chromaticity coordinates were then calculated according to the CIE 1931 definition using *x* = *X*/(*X* + *Y* + *Z*) and *y* = *Y*/(*X* + *Y* + *Z*). This procedure allowed for the quantitative assessment of color shifts among samples with different compositions.

### 2.8 Radiative Cooling Simulation

To evaluate the RC performance, spectral data were analyzed using an open‐source software, ViBA Rad [53]. The measured spectral emissivity in the MIR range was imported with atmospheric transmittance data to compute directional and hemispherical emissivity. Net cooling flux ($P_{\text{cool}}$) was computed from the radiative cooling flux of the RC surface $\left(P_{\text{rad}}\right.$), heat flux from the atmospheric radiation ($P_{\text{atm}}$), absorbed solar flux ($P_{\text{sun}}$), and heat flux from conduction and convection ($P_{\text{cc}}$), following the discrete summation forms implemented in ViBA Rad.

$P_{\text{cool}}{,P}_{\text{rad}}$, $P_{\text{atm}}$, $P_{\text{sun}}$, and $P_{\text{cc}}$ calculated using the following equations:

(1) $$P_{\text{cool}}=P_{\text{rad}}-P_{\text{atm}}-P_{\text{sun}}-P_{\text{cc}}$$

(2) $$P_{\text{rad}}=2\pi \iint \sin\;\theta \;\cos\;\theta I_{BB}\left(T,\lambda \right)\;\epsilon \left(\lambda ,\theta \right)\;d\lambda d\theta$$

(3) $$P_{\text{atm}}=2\pi \iint \sin\;\theta \;\cos\;\theta I_{BB}\left(T_{\text{amb}},\lambda \right)\;\epsilon_{\text{amb}}\left(\lambda ,\theta \right)\;\epsilon \left(\lambda ,\theta \right)\;d\lambda d\theta$$

(4) $$P_{\text{sun}}=\int I_{AM}\left(\lambda \right)\;\epsilon \left(\lambda ,\theta_{\text{sun}}\right)\;d\lambda$$

(5) $$P_{\text{cc}}=h_{\text{cc}}\;\left(T_{\text{amb}}-T\right)$$

where θ is the incident angle, T is the temperature of the RC material, $T_{\text{amb}}$ is the ambient temperature, and ϵ is the emissivity of the RC material, which were derived from the data previously obtained from a UV‐Vis‐NIR spectrophotometer and FTIR measurements. The ambient emissivity, $\epsilon_{\text{amb}}$, is defined as $1-{t\left(\lambda \right)}^{1/\cos\;\theta }$, where t(λ) is the atmospheric transmittance spectrum and $I_{BB}$ is the spectral irradiance of a black body. The convection coefficient was fixed at $h_{\text{cc}}$ = 8 Wm^−2^ K^−1^ for all simulations. The AM1.5 G reference solar spectrum (ASTM G173‐03) was used for $P_{\text{sun}}$, and blackbody radiance was computed using Planck's law. Global cooling‐flux mapping was performed using NASA LaRC POWER climatology data (surface temperature and broadband solar irradiance, July), with spectral atmospheric transmittance taken from the ViBA Rad ambient dataset to evaluate cooling potential under varying geographic and climatic conditions.

### 2.9 Outdoor Temperature Measurement

All experiments to investigate the temperature measurement of ambient, white fabric, and PVA/lignin/Al_2_O_3_ nanofibers were conducted at the rooftop of Yonsei University, Wonju. Temperature measurement was conducted by the 4‐channel handheld data logger thermometer (Omega Engineering, HH520), which served to record the real‐time temperature in the wood box. UV‐intensity measurement was conducted by the handheld weather station (ZOGLAB, HWS1000‐LTD). The wood box consists of a wood frame, aluminum foil, styrofoam, LDPE film, and acrylic. The infrared picture was taken by a thermal infrared camera (Teledyne FLIR, FLIR‐E5‐XT).

### 2.10 pH‐Responsive Sample Test

To investigate the pH‐responsive behavior, swelling tests were conducted. PVA/lignin/Al_2_O_3_ samples were immersed in buffer solutions of different pH values (pH 5 and pH 9) for 24 h, after which their swelling ratios were subsequently measured and compared. After a specific time interval, the excess surface solvent was gently removed using tissue paper, and the weight of the swollen sample (*W*_*s*_) and the weight of the dry sample *(W*_*d*_) were recorded. The same procedure was repeated until the equilibrium swelling was achieved.

The swelling ratio was calculated using the following equation:

(6) $$\text{Swelling}\;\left(g/g\right)=\frac{Ws-Wd}{Wd}\times 100\;\%$$

To investigate the pH‐dependent ionization of the samples, zeta potential variations were measured using a zeta potential analyzer (Otsuka Electronics Korea Co. Ltd., ELSZneo). In addition, to evaluate the pH‐dependent release kinetics, FLU‐loaded samples were immersed in buffer solutions of different pH values (pH 5 and pH 9), and the release kinetics were monitored over time and compared accordingly.

## 3 Results and Discussion

### 3.1 Radiative Cooling Principles: Solar Reflection and Infrared Emission

Passive daytime radiative cooling relies on a synergistic balance between minimizing solar heat gain and maximizing thermal radiation to outer space. Specifically, an ideal RC material should exhibit (i) high reflectance across the solar spectrum (0.3–2.5 μm) to suppress solar absorption and (ii) high emissivity within the MIR, especially AW (8–13 μm), where the Earth's atmosphere is transparent to thermal radiation [2, 6, 7]. These fundamental requirements are fulfilled through the integration of material composition and nanofibrous architecture in the PVA/lignin/Al_2_O_3_ composite system. A schematic illustration summarizing the coupled solar reflection and infrared emission mechanisms is provided in Figure 2a,b.

**FIGURE 2:**
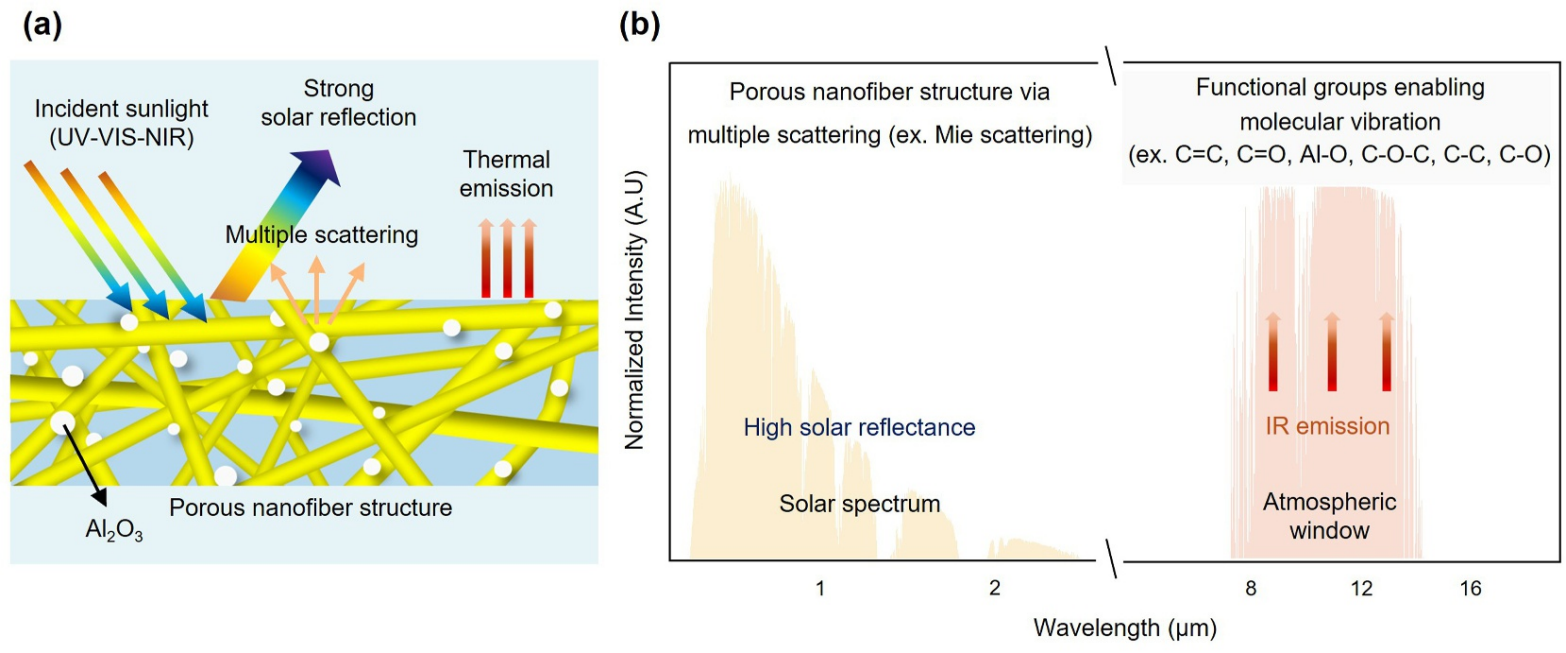
Schematic illustration of the passive daytime radiative cooling mechanism. (a) Broadband solar reflection mechanism driven by scattering from the porous nanofiber structure and Al_2_O_3_ nanoparticles. (b) Spectral coupling of solar reflection and thermal emission, highlighting high emissivity within the AW via molecular vibrations of functional groups.

To achieve effective cooling, suppressing solar absorption is a prerequisite. The electrospun nanofiber mat features a highly porous three‐dimensional architecture that creates a high density of air–fiber interfaces. These interfaces act as multiple scattering centers for incident sunlight. When scattering dominates over absorption, the optical path length within the material is significantly reduced, thereby minimizing heat gain. Furthermore, a random distribution of fiber diameters within the scale of solar wavelengths facilitates broadband Mie scattering, covering the UV‐Vis‐NIR region [32, 34, 36, 38]. The incorporation of Al_2_O_3_ nanoparticles further amplifies this effect, as their high refractive index relative to the polymer matrix increases the overall scattering cross‐section [44, 45, 46, 47]. Consequently, although lignin intrinsically absorbs sunlight, this absorption is effectively mitigated by the combined effects of PVA‐based nanofiber‐induced multiple scattering and dielectric scattering from the Al_2_O_3_ nanoparticles.

Complementing high solar reflection, efficient heat dissipation requires strong thermal emission within the AW. Molecular vibrational modes associated with functional groups (such as C=O, C–O‐C, Al‐O, C‐C) provide strong absorption and emission bands in the MIR region. In our composite, the abundant functional groups in PVA, lignin, and Al_2_O_3_ contribute to strong vibrational absorption within the 8–13 μm range. These molecular vibrations enable high emissivity, allowing thermal radiation to escape directly into cold outer space. This synergistic integration of broadband solar reflection, driven by the porous nanofiber architecture and high‐refractive‐index Al_2_O_3_ nanoparticles, and strong MIR emission originating from the molecular vibrations of the polymer matrix fundamentally enables the sub‐ambient cooling performance.

### 3.2 Electrospinning of PVA/Lignin/Al_2_O_3_ Nanofiber Composites

PVA/lignin/Al_2_O_3_ nanofiber composites were fabricated via an electrospinning process. The fabrication process for the crosslinked PVA/lignin/Al_2_O_3_ nanofiber composites is shown in Figure 3a. PVA, a water‐soluble and biocompatible polymer, was selected as the primary matrix due to its strong vibrational modes in the MIR region, which contribute to emissivity. The abundant hydroxyl groups in PVA also enable physical or chemical crosslinking, allowing the formation of hydrogel networks that provide structural stability in nanocomposite fabrication. Lignin, a renewable and biodegradable biopolymer, was incorporated as a complementary component. Its ionizable functional groups (–OH, –COOH) enable pH‐responsive swelling behavior, whereas its intrinsic MIR absorption further enhances RC. In addition, the reactive sites in lignin facilitate hydrogel network formation together with PVA, improving the versatility and stability of the composite system. To further enhance optical performance, Al_2_O_3_ nanoparticles were introduced to increase solar reflectance, compensating for the intrinsic dark coloration of lignin. Citric acid was employed as a crosslinking agent, enabling the formation of ester bonds between hydroxyl and carboxyl groups during thermal treatment. This step improved water stability in aqueous environments while maintaining sufficient flexibility for subsequent performance evaluation.

**FIGURE 3:**
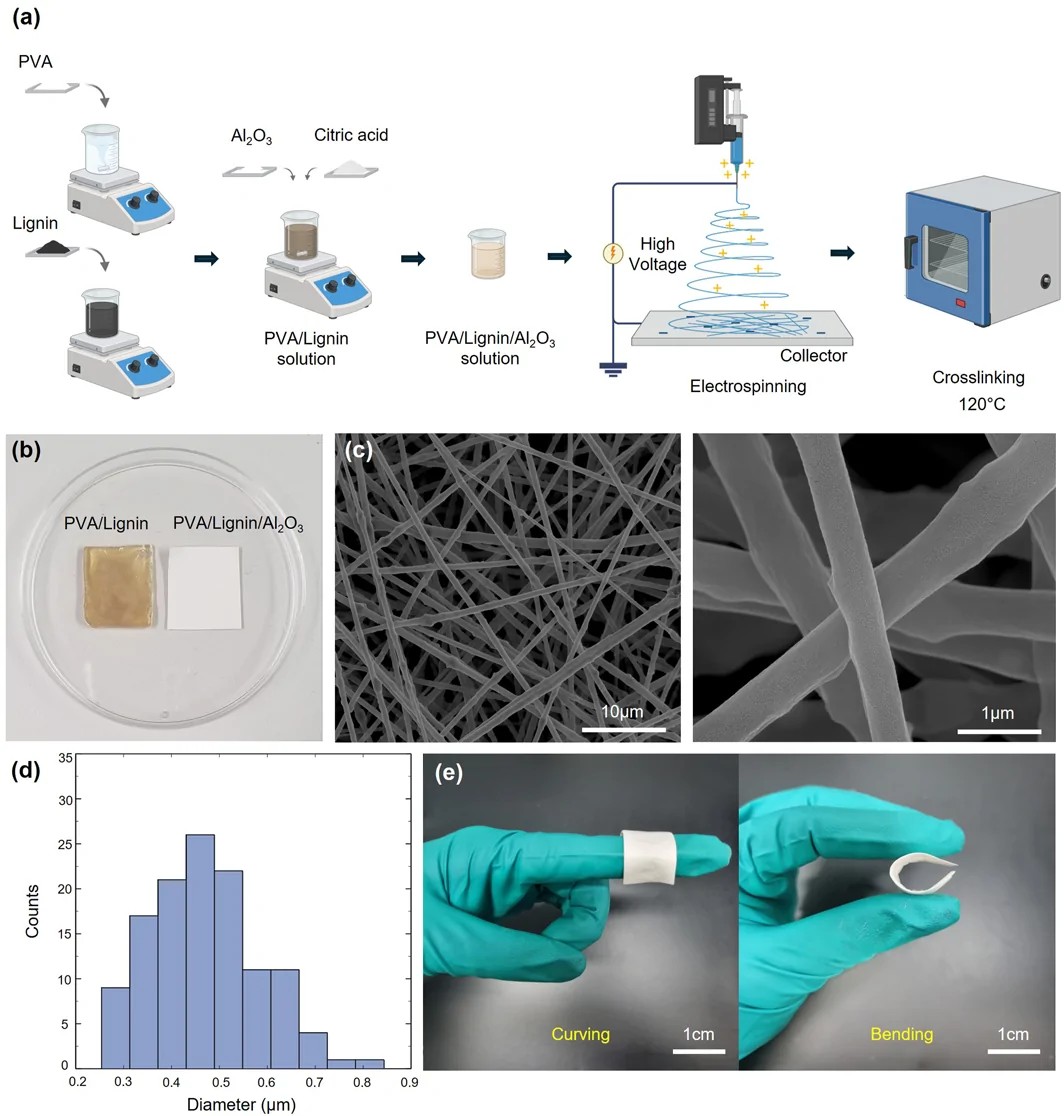
Morphology and fabrication of PVA/lignin/Al_2_O_3_ nanofibers. (a) Schematic of the electrospinning fabrication (created with BioRender.com). (b) A photograph of fabricated PVA/lignin sample and PVA/lignin/Al_2_O_3_ nanofibers sample, and (c) SEM images of the fabricated PVA/lignin/Al_2_O_3_ nanofibers sample. (d) Distribution of fiber diameters. (e) Photographs of a 1 cm × 2.5 cm‐sized sample to show its flexibility.

As noted earlier, the practical use of lignin‐based materials has been hindered by their inherently dark color. To address this limitation, solar reflectance should be increased while preserving the functional advantages of lignin [41]. To investigate the effects of Al_2_O_3_ incorporation and the fabrication method, two samples with the same PVA:lignin ratio were prepared for comparison. The PVA/lignin film sample was fabricated by film casting, whereas the PVA/lignin/Al_2_O_3_ nanofiber sample was produced through electrospinning (Figure 3b). Despite incorporating lignin, the PVA/lignin/Al_2_O_3_ nanofiber sample appeared brighter than the PVA/lignin film sample. Notably, the addition of Al_2_O_3_ and the electrospinning process resulted in a visibly whiter sample, thereby suggesting an enhancement in solar reflectance. As shown in Figure 3c, SEM images further confirmed the successful formation of uniform, bead‐free nanofibers with a three‐dimensional porous architecture. Such porous morphology is expected to enhance optical performance by providing multiple scattering interfaces within the fibrous network. Moreover, the interconnected pores are irregular in shape and well‐dispersed throughout the nanofibrous mat. Quantitative porosity analysis based on ImageJ revealed a porosity of approximately 41.47%, indicating a high density of internal air–fiber interfaces, thereby promoting multiple light scattering within the fibrous network. As shown in Figure 3d, ImageJ analysis revealed a fiber diameter range of 0.2–0.8 μm, which is suitable for Mie scattering of incident solar radiation, thereby reducing solar absorption and enhancing RC performance.

The PVA/lignin/Al_2_O_3_ nanofiber composites also exhibited notable mechanical flexibility (Figure 3e). These samples could be bent and curved without visible structural damage, indicating that the electrospun network retained its integrity after crosslinking. Such flexibility is beneficial for applications requiring conformal contact with irregular surfaces or integration onto various substrate types.

### 3.3 Effect of Crosslinking on the Properties of PVA/Lignin/Al_2_O_3_ Nanofiber Composites

Mechanical strength and water resistance are critical requirements for nanofiber composites in practical use. However, electrospun PVA/lignin/Al_2_O_3_ nanofibers are limited by the inherent water solubility of PVA and lignin, which reduces both mechanical integrity and stability in aqueous environments. To address these limitations, citric acid was introduced as a crosslinker, forming ester bonds between hydroxyl and carboxyl groups. This approach improved load transfer through covalent linkages while simultaneously reducing solubility, thereby enhancing both mechanical robustness and water stability. To assess the effect of crosslinking on the mechanical performance of the nanofiber composites, tensile strength measurements were conducted before and after the crosslinking process (Figure 4a). It revealed improvements in the mechanical properties after crosslinking, with the ultimate tensile strength increasing from 8.63 MPa at 78.17% strain in the uncrosslinked state to 11.57 MPa at 104.26% strain after crosslinking, whereas the elongation at break increased from 98.32% to 111.09%. These gains arise from covalent bonds between polymer chains, which enhance load transfer and promote uniform stress distribution, while preserving ductility. The resulting balance of strength and deformability supports use in applications demanding conformal contact with nonplanar surfaces across diverse functional and structural systems.

**FIGURE 4:**
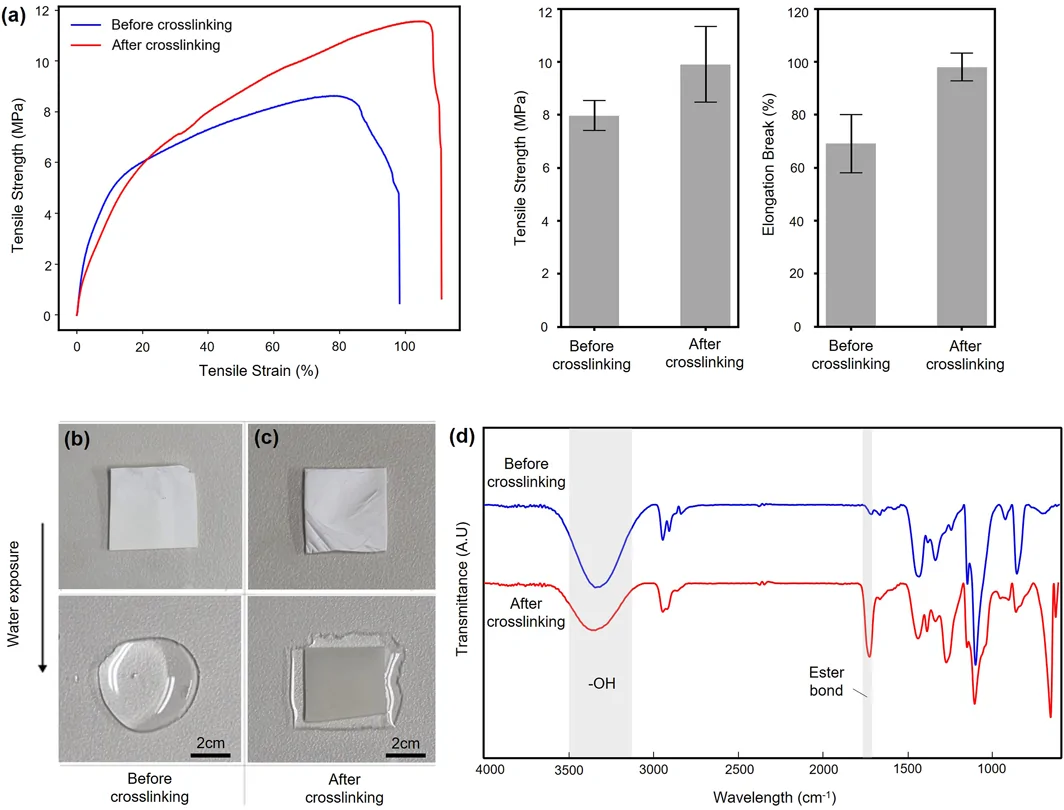
Mechanical and chemical stability of crosslinked PVA/lignin/Al_2_O_3_ nanofibers. (a) Tensile stress–strain curves (left) and tensile strength (middle) and elongation at break (right) of PVA/lignin/Al_2_O_3_ nanofibers before and after crosslinking. (b) Before crosslinking and (c) after crosslinking, highlighting improved water resistance and structural stability of the crosslinked samples upon water exposure. (d) FTIR spectra of the uncrosslinked and crosslinked PVA/Lignin/Al_2_O_3_ nanofibers.

Water stability was further assessed by exposing the nanofiber mats to aqueous environments. As shown in Figure 4b,c, the image on the left illustrates the sample before crosslinking, whereas the image on the right shows the sample after crosslinking. When the noncrosslinked sample was exposed to water, the nanofibers rapidly dissolved, causing the disappearance of the sample (Figure 4b). Conversely, the crosslinked sample maintained its form upon water exposure (Figure 4c). This observation confirms a substantial improvement in the water stability of the nanofibers, which contribute to their potential for application in diverse fields. To characterize the occurrence of crosslinking in the fabricated samples, FTIR spectral analysis was employed to monitor the chemical changes before and after the crosslinking (Figure 4d). The blue spectrum corresponds to the uncrosslinked sample, whereas the red spectrum represents the data obtained from the sample crosslinked with the addition of citric acid. A notable change was observed in the ‐OH group region, typically distributed between 3500 cm^−1^ and 3200 cm^−1^. Before the crosslinking, a strong and broad peak was evident around 3300 cm^−1^, characteristic of the abundant hydroxyl groups in PVA and lignin. After the crosslinking, a significant reduction in the intensity of this peak was observed, clearly indicating a decrease in the concentration of ‐OH groups due to their involvement in the crosslinking reaction. Furthermore, the red spectrum distinctly shows the formation of a new peak in the region of 1700 cm^−1^ to 1750 cm^−1^, which is characteristic of the stretching vibration of the ester (C=O) bond. The emergence of this ester bond peak provides strong evidence that crosslinking has successfully occurred within the nanofibers.

### 3.4 Effect of PVA:Lignin Ratio and Al_2_O_3_ on Solar Reflectance and Brightness

The optical characteristics of the fabricated PVA/lignin/Al_2_O_3_ nanofibers were investigated, focusing on color variation and solar spectrum reflectance as functions of the PVA:lignin ratio and the presence of Al_2_O_3_. Figure 5a presents visual images illustrating the color changes observed in the samples under different compositional conditions. The top‐left image in Figure 5a shows a sample prepared with a PVA:lignin ratio of 8:2, incorporating Al_2_O_3_. At this specific ratio, the sample surface exhibits a noticeable brownish hue. In contrast, samples fabricated with higher PVA concentrations, specifically at the PVA:lignin ratio of 9.5:0.5, appear significantly brighter and whiter. The influence of Al_2_O_3_ on their reflectance is presented in Supporting Information S1: Figure S1, showing an enhancement of reflectance across the solar spectrum. Despite the identical polymer ratio, a difference in reflectance is evident based on the presence or absence of Al_2_O_3_, highlighting its crucial role in enhancing reflectance. This enhancement is primarily attributed to the refractive index difference between the nanoparticles and the matrix. These high‐refractive‐index nanoparticles exhibit the dielectric behavior across the solar spectrum and a huge refractive index contrast with the medium, thereby increasing the overall scattering cross‐section of the composite and enhancing broadband solar reflection.

**FIGURE 5:**
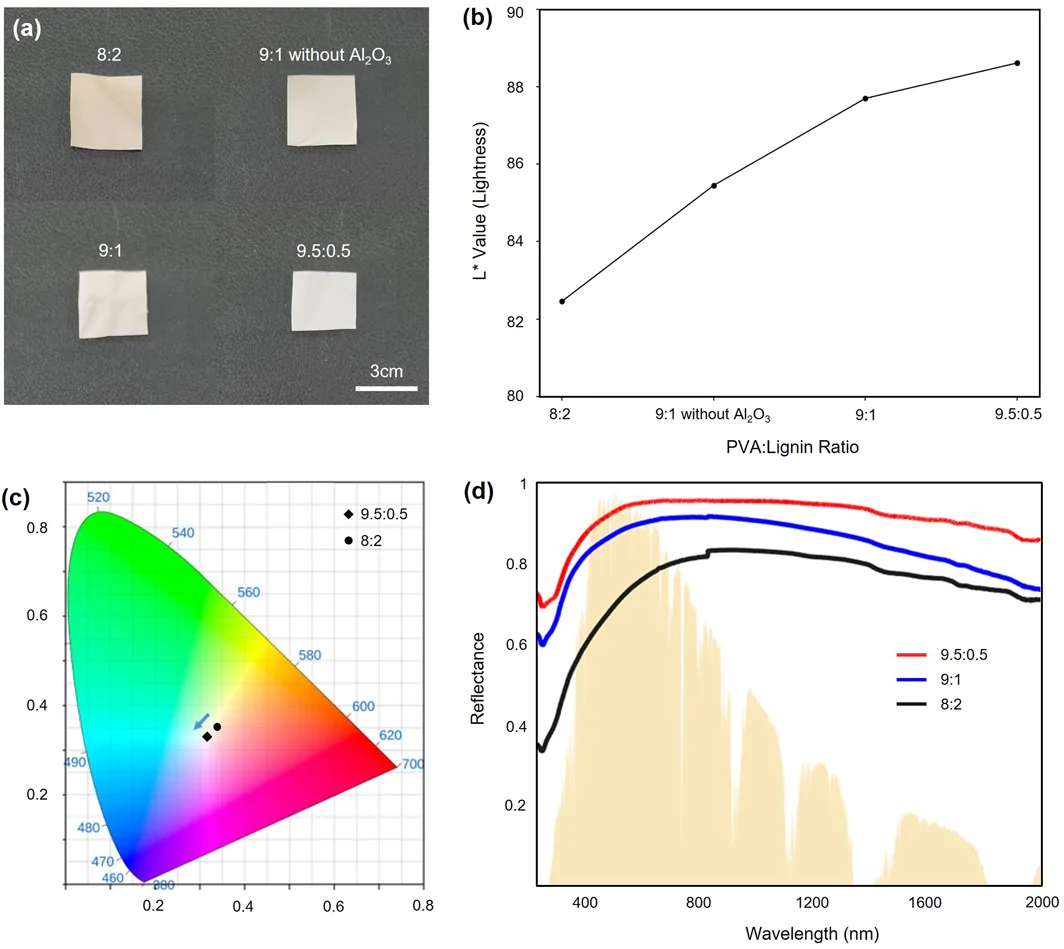
Optical characteristics of nanofibers with different compositional conditions. (a) Color change of nanofibers with varying PVA:lignin ratios. Top left: 8:2 (with Al_2_O_3_), top right: 9:1 (without Al_2_O_3_), bottom left: 9:1 (with Al_2_O_3_), bottom right: 9.5:0.5 (with Al_2_O_3_). (b) Lightness changes of nanofibers by PVA:lignin ratios: 8:2 (with Al_2_O_3_), 9:1 (without Al_2_O_3_), 9:1 (with Al_2_O_3_), 9.5:0.5 (with Al_2_O_3_). (c) Color shift in the CIE chromaticity. (d) Reflectance of nanofibers with different PVA:lignin ratios. Red: 9.5:0.5 (with Al_2_O_3_), blue: 9:1 (with Al_2_O_3_), black: 8:2 (with Al_2_O_3_).

As shown in Figure 5b, the L* (lightness), a perceptual metric in which 0 denotes black and 100 denotes diffuse white under a fixed white point, increased monotonically with higher PVA content (82.5 at 8:2 to 88.6 at 9.5:0.5). Such an increase in lightness is advantageous for RC applications, as brighter surfaces generally exhibit higher solar reflectance. This trend is consistent with the shift of chromaticity coordinates toward the achromatic point, indicating reduced coloration and increased perceived brightness (Figure 5c). As the PVA:lignin ratio increased from 8:2 to 9.5:0.5, the CIE chromaticity coordinates moved toward the near‐white region, approaching the achromatic point (*x* ≈ 0.33, *y* ≈ 0.33). Specifically, the 8:2 sample exhibited coordinates of (0.339, 0.353), whereas the 9.5:0.5 sample shifted to (0.317, 0.334), indicating a movement of Δ*x* = −0.022 and Δ*y* = −0.019 toward the achromatic point. The 9.5:0.5 sample showed the smallest chromaticity distance from the achromatic point, reflecting the most neutral tone. This trend is consistent with the observed increase in L* values and can be attributed to the combined effects of reduced lignin coloration and enhanced light scattering from Al_2_O_3_, resulting in a perceptible enhancement in whiteness. Figure 5d displays the reflectance data across a range of wavelengths for nanofibers with varying PVA:lignin ratios and Al_2_O_3_ content. Among these compositions, the nanofiber with a PVA:lignin ratio of 9.5:0.5 (red line) exhibited the highest reflectance, indicating that the optical properties can be effectively tuned through compositional control.

### 3.5 Radiative Cooling Performance

To characterize the RC properties of the fabricated material, its reflectance in the solar spectrum range was measured using a UV‐Vis‐NIR spectrophotometer, and its emissivity in the MIR region was determined using FTIR spectroscopy. The results showed an average reflectance of 0.88 across the solar spectrum, with a particularly excellent average reflectance of 0.92 in the visible region (Figure 6a). This strong visible reflectance is attributed to the Mie scattering from the porous hierarchical nanostructure of the PVA/lignin/Al_2_O_3_ nanofibers and the Al_2_O_3_ nanoparticles. This characteristic is essential for reducing solar heat absorption, which enables effective daytime RC. Furthermore, the emissivity in the MIR region, specifically within AW, exhibited an average value of 0.97. These optical characteristics indicate that the fabricated material possesses spectral properties desired for effective RC.

**FIGURE 6:**
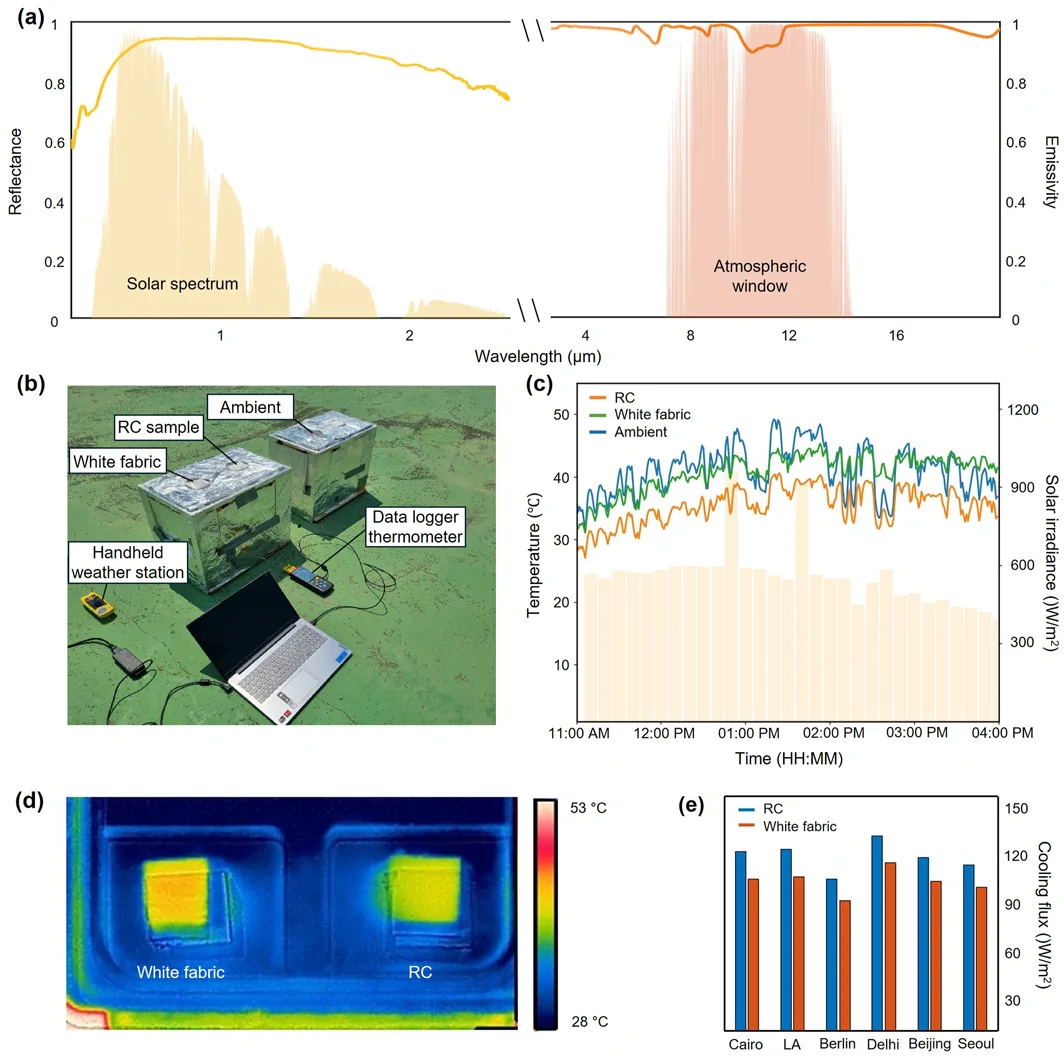
Radiative cooling performance of PVA/lignin/Al_2_O_3_ nanofiber composites. (a) Solar reflectance and mid‐infrared emissivity spectra of the fabricated PVA/lignin/Al_2_O_3_ nanofiber composites. (b) Outdoor experimental setup for temperature measurement. (c) Real‐time outdoor temperature of the RC film, white fabric, ambient and solar irradiance measured during daytime. (d) Infrared thermal images comparing the RC film and the white fabric. (e) Comparison of the cooling flux in the selected major cities between the RC nanofibers (blue bar) and white fabric (red bar).

Subsequently, an outdoor temperature measurement experiment was conducted to evaluate the actual temperature reduction capability of the fabricated material. To minimize the influence of heat transfer from the ground, thermal conduction, and convection, a custom measurement apparatus was constructed using a wood frame, aluminum foil, styrofoam, acrylic, and PE film (Figure 6b). For temperature monitoring, three comparative samples included commercial white fabric, the fabricated RC material, and the ambient air temperature. A comparative analysis revealed that our RC film consistently maintained a lower temperature than both the ambient air and the white fabric control (Figure 6c). A comparison between the RC film and white fabric temperatures indicated a significant average difference of 5.6°C and a maximum difference of 9.2°C. Moreover, the RC film exhibited an average temperature difference of 5.4°C and a maximum temperature difference of 8.9°C compared to the ambient temperature. The solar irradiance data represented as a yellow shade was obtained from the Korea Institute of Energy Research, whereas the UV intensity was measured on‐site using a handheld weather station (Supporting Information S1: Figure S2). The fluctuations observed in the UV intensity data are attributed to intermittent cloud cover (Supporting Information S1: Figure S3), reflecting the dynamic nature of real outdoor conditions. Despite these variations caused by intermittent cloud cover, the RC film consistently maintained sub‐ambient temperatures, highlighting its reliability under fluctuating environmental conditions. To further evaluate the outdoor performance under different humidity environments, the cooling capability was analyzed with respect to varying relative humidity (RH). As shown in Supporting Information S1: Figure S4, the RC film maintained consistent sub‐ambient cooling across different RH levels, confirming its robustness against atmospheric moisture.

Figure 6d presents thermal infrared images, captured by a thermal infrared camera (with an emissivity setting of 0.95), illustrating the surface temperatures of the RC material and the white fabric. A visual comparison of the infrared images' color scales confirmed that the RC film maintained a relatively lower surface temperature compared to the white fabric. Collectively, these findings unequivocally demonstrate the outstanding RC performance of the fabricated PVA/lignin/Al_2_O_3_ nanofiber composites.

To evaluate the cooling performance on a global scale, the global cooling flux was calculated based on the measured optical spectral data of the PVA/lignin/Al_2_O_3_ nanofiber composites. The cooling fluxes of the fabricated RC sample and white fabric were calculated and compared with those of a white fabric for six selected major cities (Figure 6e). The results consistently demonstrated that the RC sample achieved significantly superior cooling performance compared to the white fabric in all six cities. Moreover, the global cooling fluxes were calculated and mapped to visualize their distribution worldwide (Supporting Information S1: Figure S5). The results revealed predominantly positive cooling flux values with a maximum of 155.35 W m^−2^. These indicate that the radiative cooling performance of the fabricated material remains highly effective even under intense solar irradiance and can deliver meaningful cooling benefits on a global scale.

### 3.6 pH Responsiveness Test

To investigate the pH‐responsiveness of the fabricated PVA/Lignin/Al_2_O_3_ nanofiber composites, a series of characterizations was conducted, encompassing swelling behavior, zeta potential analysis, pH‐dependent release of a model compound, and RC performance under controlled outdoor conditions (Figure 7). The swelling ratio of the nanofiber hydrogel was measured after 24 h of immersion in pH 5 and pH 9 buffer solutions (Figure 7a). The hydrogel exhibited a swelling ratio of 829.01% at pH 5, which significantly increased to 1206.57% at pH 9. This increase of approximately 45.5% is attributed to enhanced electrostatic repulsion among ionized functional groups. At elevated pH, deprotonation of phenolic hydroxyl and carboxyl groups generates negative charges, resulting in increased electrostatic repulsion within the polymer network and subsequent expansion, which facilitates greater water uptake. This mechanism is further supported by zeta potential measurements (Figure 7b). At pH 5, the zeta potential was −3.88 mV, becoming more negative with increasing pH and reaching −8.79 mV at pH 9. This trend reflects the increased degree of ionization of acidic functional groups, aligning with the observed swelling behavior.

**FIGURE 7:**
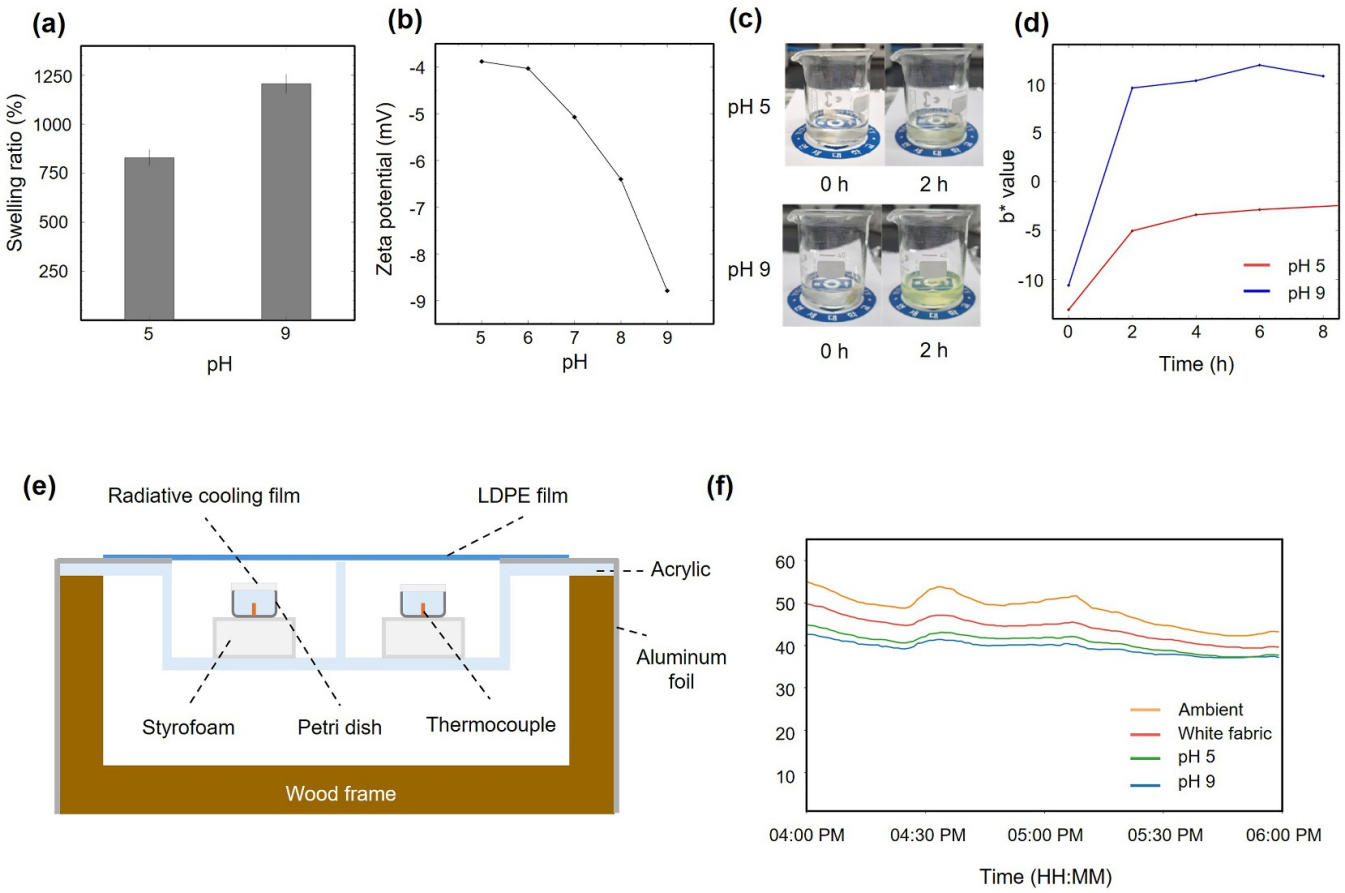
pH‐responsive behavior of PVA/lignin/Al_2_O_3_ nanofiber hydrogel. (a) Swelling ratio at pH 5 and pH 9. (b) Zeta potential as a different pH. (c) Visual observation of FLU release behavior from the nanofiber hydrogel at pH 5 (top) and pH 9 (bottom). (d) Change in b* value, analyzed with ImageJ, to represent fluorescein release over time (red: pH 5, blue: pH 9). (e) Schematic of outdoor temperature measurement setup for the pH environment. (f) Real‐time outdoor temperature comparison under varying pH conditions: orange (ambient), red (white fabric), green (RC with pH 5), and blue (RC with pH 9).

The pH‐responsive swelling feature of the nanofiber composites leads to distinct release kinetics under different pH environments, which is essential for extending their applicability to other stimuli‐responsive or controlled‐release systems. To visually evaluate the pH‐responsive release behavior, FLU was incorporated into the nanofibers as a yellow dye indicator, after which the release was monitored under both pH 5 and pH 9 conditions (Figure 7c). After 2 h, the pH 9 sample displayed a pronounced color change indicative of FLU release, whereas the pH 5 sample exhibited less noticeable visible change. This observation was further quantified by analyzing the change in the b* value from the CIE LAB color space over time using ImageJ software (Figure 7d). The b* value, representing the yellow–blue color axis, began to diverge significantly between pH 5 and pH 9 after 2 h, confirming that the release behavior is highly pH‐sensitive, which is desirable for various stimuli‐responsive and functional material applications.

To evaluate whether the RC performance of the hydrogel is affected by pH, an outdoor temperature measurement setup was implemented under various pH conditions (Figure 7e). The system employed thermocouples housed in sealed petri dishes beneath RC films subjected to either pH 5 or pH 9. Both RC hydrogels achieved substantial sub‐ambient cooling compared to the ambient temperature and a white fabric control (Figure 7f). Specifically, RC hydrogels exposed to pH 5 and pH 9 showed average cooling of 8.4°C and 9.9°C relative to ambient conditions, and 3.7°C and 5.1°C relative to the white fabric, respectively. Notably, the temperature difference between the pH 5 and pH 9 samples was relatively minor (average 1.5°C), suggesting that the intrinsic optical properties of the material dominate the cooling behavior rather than the external pH environment. These results indicate that the composite maintains its RC performance even in hydrogel states under different pH conditions. Overall, these results highlight the multifunctionality of the developed PVA/lignin/Al_2_O_3_ nanofiber hydrogel, demonstrating both effective passive cooling and pH‐responsive release behavior, thereby positioning it as a promising platform for intelligent and sustainable material systems.

## 4 Conclusion

In summary, this study demonstrates a multifunctional nanofiber composite that integrates radiative cooling and pH‐responsiveness through the combination of PVA, lignin, and Al_2_O_3_. This dual functionality is enabled by materials that both respond to pH variations and exhibit strong emission within the atmospheric window. Importantly, this work provides a conceptual and experimental foundation for expanding radiative cooling technologies from static thermal regulation toward dynamically responsive systems. This integration enables potential deployment in diverse applications, ranging from intelligent food packaging and environmental monitoring to biomedical systems requiring pH‐responsive wound dressings and controlled drug delivery, where simultaneous responsiveness and energy efficiency are desired.

## Author Contributions

**Seokgyu Kwon:** conceptualization, methodology, investigation, writing – original draft. **Minseo Jeong:** investigation, validation. **Changhwan Hyeon:** investigation. **Juhoon Baek:** investigation. **Minkyung Kim:** supervision, funding acquisition, writing – review and editing. **Dasol Lee:** supervision, funding acquisition, writing – review and editing. All authors have read and agreed to the published version of the manuscript. All authors have accepted responsibility for the entire content of this manuscript and consented to its submission to the journal, reviewed all the results and approved the final version of the manuscript.

## Funding

D. Lee acknowledges the National Research Foundation (NRF) grants (RS‐2025‐00556856) funded by the Ministry of Science and ICT (MSIT) of the Korean government. M. Kim acknowledges the InnoCORE program of the Ministry of Science and ICT (GIST InnoCORE KH0860).

## Conflicts of Interest

The authors declare no conflicts of interest.

## Supporting information


Supporting Information S1

## Data Availability

The data that support the findings of this study are available from the corresponding author upon reasonable request.
