# Supplementary material for: Multifunctional Nanofiber Composites Integrating pH‐Responsiveness and Passive Daytime Radiative Cooling
Source: Nanophotonics. 2026 Apr 1;15(7):e70036. doi: 10.1002/nap2.70036 (PMC13642550; doi:10.1002/nap2.70036)
Supplement: Supplementary file 1 [file NAP2-15-e70036-s001.docx]

**Supplementary Material**

Seokgyu Kwon, Minseo Jeong, Changhwan Hyeon, Juhoon Baek, Minkyung Kim^*^, and Dasol Lee^*^

Multifunctional Nanofiber Composites Integrating pH-Responsiveness and Passive Daytime Radiative Cooling

**Keywords:** radiative cooling; electrospinning; pH-responsiveness; nanofiber composites.

***Corresponding author:** **Dasol Lee**, Department of Biomedical Engineering, Yonsei University Wonju 26493, Republic of Korea. E-mail: [dasol@yonsei.ac.kr](mailto:dasol@yonsei.ac.kr)

***Corresponding author: Minkyung Kim**, Department of Mechanical and Robotics Engineering, Gwangju Institute of Science and Technology (GIST), Gwangju 61005, Republic of Korea. E-mail: m.kim@gist.ac.kr

**Seokgyu Kwon, Minseo Jeong, Changhwan Hyeon**, Department of Biomedical Engineering, Yonsei University, Wonju 26493, Republic of Korea. E-mail: [hk2718@yonsei.ac.kr](mailto:hk2718@yonsei.ac.kr)

**Juhoon Baek**, Department of Mechanical and Robotics Engineering, Gwangju Institute of Science and Technology (GIST), Gwangju 61005, Republic of Korea.

**Figure S1**: Reflectivity enhancement by Al₂O₃ incorporation in PVA/Lignin nanofibers.

**Figure S2**: Real-time variation of UV intensity during outdoor measurement.

**Figure S3**: Temporal variation of total cloud cover expressed on a 0–10 scale.

**Figure S4**: Outdoor radiative cooling performance under different relative humidity conditions.

**Figure S5**: Global cooling fluxes calculated and mapped using ViBA-Rad.
